# A new treatment for cervical dizziness^☆^

**DOI:** 10.1016/j.bjorl.2023.101321

**Published:** 2023-09-11

**Authors:** Cesar Bertoldo Garcia, Nedison Gomes Paim Alves, Roseli Saraiva Moreira Bittar

**Affiliations:** a Faculdade de Medicina da Universidade de São Paulo (FMUSP), Pós-Graduação em Medicina, São Paulo, SP, Brazil; b Faculdade de Medicina da Universidade de São Paulo (FMUSP), Pós-Graduação em Ciências, São Paulo, SP, Brazil; c Hospital das Clínicas da Faculdade de Medicina da Universidade de São Paulo (HC/FMUSP), Departamento de Otorrinolaringologia, Setor de Otoneurologia, São Paulo, SP, Brazil

**Keywords:** Dizziness, Neck pain, Proprioception, Vertigo, Thermic treatment

## Abstract

**Objectives:**

To verify the rates of heat effectiveness generated by Carbon Nanotubes (CNT) in the resolution of dizziness in individuals diagnosed with Cervical Dizziness (CD).

**Methods:**

Randomized clinical trial was performed and samples from volunteers diagnosed with CD, divided into a Study Group (SG) and Control Group (CG). Patches containing CNT with thermotherapeutic effect were used in SG and inert patches in the CG. Treatment was performed over 30 days and measures of pain, dizziness and asymmetry of cervical muscle tension were evaluated over 60 days.

**Results:**

Pain, dizziness and asymmetry of cervical muscle tension were reduced in the SG. The variables analyzed in this study did not change in the CG.

**Conclusion:**

Our results show that the therapeutic effect of heat was effective in the treatment of patients diagnosed with CD. We could also observe a clear interrelationship between pain and dizziness symptoms with the asymmetry of cervical muscle tension.

**Level of evidence:**

1B.

## Highlights

• Dizziness is related to pain in patients diagnosed with Cervical Dizziness (CD).
• Dizziness is related to muscle tension asymmetry in the CD.
• The new treatment with carbon nanotubes is effective in treating patients with CD.

## Introduction

Cervical origin accounts for 7.5% of dizziness etiologies; it impacts quality of life and is a growing concern within the realm of community health.^1^ Its effect can be debilitating, causing mobility impairment, emotional disturbances, and reduced productivity.^2^ The diagnosis of Cervical Dizziness (CD) is challenging, as its symptoms overlap with other conditions, compounded by the absence of specific clinical or laboratory tests. Consequently, the described aspects require a meticulous and differentiated approach to CD.^3^

By definition, CD is a clinical syndrome characterized by the presence of cephalic signs and symptoms originating in the cervical segment.^4^ The observed symptoms are attributed to incongruence between erroneous information arising from cervical proprioceptors, present in tense muscles during cephalic movement, and the neural map previously established in the Central Nervous System (CNS).^5^ This information discrepancy leads to disorientation, fluctuation, or imbalance, lasting from minutes to hours,^3^, ^6^ in addition to visual alterations and falls.^7^

Advancements in understanding the mechanisms involved in CD have facilitated the development of potential therapies. The use of manual therapy with positive outcomes in treatment has been described.^8^ Nonetheless, studies presenting proven effective long-term treatment options remain limited.^9^ In this context, the recent utilization of nanotechnology, involving the use of heat-generating coils in the form of patches, designated as Helical®, holds great promise. This is a mechanical device composed of an adhesive-coated active central disc, measuring 2 cm in diameter. The active central region contains a coil composed of Carbon Nanotubes (CNT) arranged in a spiral, which, upon adhering to the skin, absorbs electromagnetic waves from tense musculature and dissipates them in the form of heat. The emitted heat raises the local temperature by 0.7–1.2 °C.^10^, ^11^ It is documented that the local metabolic rate increases by 10%–13% as a consequence of each Celsius degree elevation.^12^ The increase in metabolic function provides better nutrient supply, reduction of the inflammatory process, and elimination of toxins, resulting in muscle relaxation, pain relief, and localized stiffness alleviation. The effectiveness of the CNT in the treatment of cervical dizziness has also been proven good results in a study without a Control Group.^13^

This investigation had as its primary objective to analyze the role of CNT in relieving cervical pain and dizziness and to provide a new method to treat these disorders. In addition, the secondary objective was to evaluate how muscle tension asymmetry is associated with cervical pain and dizziness in patients diagnosed with CD.

## Methods

This is a randomized, double-blind clinical trial. The research team consisted of two physicians, a coordinator, an executor, and a physiotherapist. Participant randomization into Study Group (SG) and Control Group (CG) was performed based on a randomization list by the coordinating physician. The executing physician, physiotherapist, and participants were blinded to treatment allocation, which involved the application of patches with active CNT or placebo. Placebo patches, inert in nature, were employed in the study and prepared to be visually indistinguishable in appearance, form, and packaging. Envelopes containing the patches for application were coded to conceal their identity from participants, the physiotherapist, and the executing physician. Furthermore, the subtle thermal alteration ensures that both the SG and the CG do not perceive any difference in the adhesive applied to their skin. The coordinating physician monitored the integrity of blinding throughout the study, ensuring the objectivity and reliability of results. This approach was fundamental, aiming to minimize potential bias in the study and thereby ensuring the validity of the obtained outcomes.

The patients were previously submitted to anamnesis, complete otoneurological examination, serological and electrophysiological tests, diagnostic imaging, and psychological evaluation. The diagnosis was based on dizziness characterized as imbalance, instability, or vertigo, necessarily related to the onset of cervical pain. No other vestibular diseases were identified, including vestibular migraine. All participants underwent cervical X-ray and computed tomography, or magnetic resonance imaging of the cervical spine were used when available.

The inclusion criteria were diagnosis of CD, normal otoneurological examination findings, signing the informed consent form, no cervical radiological changes other than those attributable to ageing. The exclusion criteria were active vestibular disease; traumatic neck injury; neurological, rheumatic, or orthopaedic diseases not attributable to ageing (fractures, tumours) and/or those for which surgery is indicated; somatoform diseases; or cognitive limitations.

Data from a preliminary pilot study provided the basic scenario for calculating the experiment’s sample size. A comparison of two means was performed, considering a standard deviation of 1.7, in a superiority test with a significance level of 5% and a power of 80%. A sample size of 16 patients per group was obtained. After patient recruitment commenced, a preliminary analysis was conducted, resulting in a statistically significant difference. The study was concluded with 20 patients: 15 in the SG and five in the CG.

The groups received ten patches each. They were placed in the posterior cervical region: two in the suboccipital region, two around the fifth and sixth vertebrae, two in the transition region between the neck and shoulder, and the others in the region of greatest pain reported by the patient. The same physical therapist performed all assessments.

All patients followed the same steps and procedures, except for the type of intervention, whether active or placebo patches. The complete protocol lasted 60 days. During the intervention period, the patches were initially applied and subsequently replaced on day 15. Particularly, on day 30, the patches were removed, and patients remained patch-free for the ensuing 30 days to discern post-treatment effects. This strategic approach was guided by the findings of a prior study, which indicated a reduction in effectiveness of the patches after two weeks of continuous use.^13^ Four face-to-face visits were required:

Day 1 — the volunteers answered the Visual Analogue Scale (VAS) questionnaires. Subsequently, the physiotherapist accessed the cervical region. Muscle and/or joint tender points were identified. Muscle tension was measured in the posterior region of the neck using a Neutone® device in its upper, middle, and lower portions. Each region was measured three consecutive times and the weighted mean was calculated. The patches were placed, and patients were instructed to remain with the patches for the entire time and replace them if they fell out and not to use pain medication.

Day 15 — first return. Participants were questioned about neck pain and dizziness preceding period using the VAS. Muscle tension was measured. Side effects or medication use were recorded. Patches were replaced with new ones.

Day 30 — procedures performed in the previous appointment were repeated and the data were registered. The patches were removed.

Day 60 — in the fourth and final evaluation, one month after the intervention, VAS was administered, and the measurement of muscle tension was performed.

## Results

Of the 20 selected patients, only one patient in the SG reported localized pruritus as a side effect. Due to the mild nature of the reported complaint, there was no interruption of treatment until the completion of the research.

### Statistical analysis

Study variables showed a normal distribution and were compared at baseline using the Student’s *t*-test. Imaging tests were compared with the Fisher’s test.

Muscle tension measurements related to pain and dizziness over time were evaluated by a mixed linear model. The model factors included patient, time, and treatment. The patient was considered a random factor. Therapy and time were fixed effects. The model also evaluated the treatment-time interaction. There were repeated measurements for each patient. The Restricted Maximum Likelihood method was used for adjustments. Significance levels of multiple comparisons were corrected using the Tukey-Kramer procedure.

The study considered a significance level of 5% (*α* = 0.05) and a Confidence Interval of 95%.

### Epidemiology

The mean age of the SG was 63.7 ± 9.4 years, and the mean age of the CG, 64.4 ± 3.8 years. Most (18) participants were women (90%). The groups had no different study variables at baseline (Table 1).

**Table 1:** Epidemiology of participants.

Group	Mean age (years)	Standard deviation	Gender (female)	Gender (male)	Participants
SG	63.7	9.4	14	1	15
CG	64.4	3.8	4	1	5

CS, Control Group; SG, Study Group.

### Visual Analogue Scale

**Figure 1:**
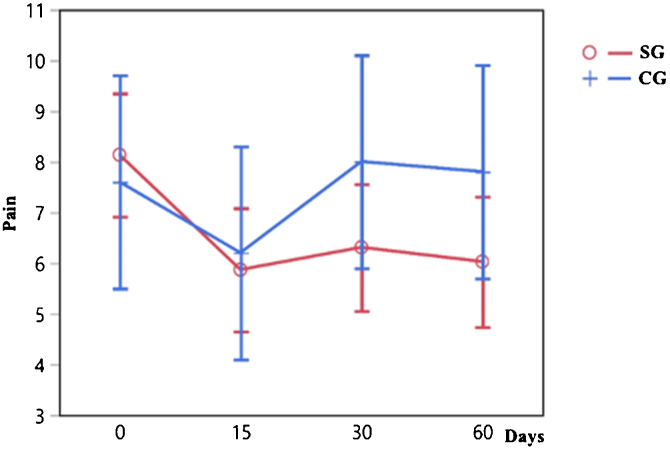
Progression of pain indices by VAS throughout the study, individualized for the SG (red line) and CG (blue line) by the LSM.

There is a difference in the indices obtained by the VAS for pain between 0 and 15, 30 and 60 days in the SG (*p* < 0.0001), which did not occur in the CG (*p* = 0.1060), as shown in Fig. 1.

**Figure 2:**
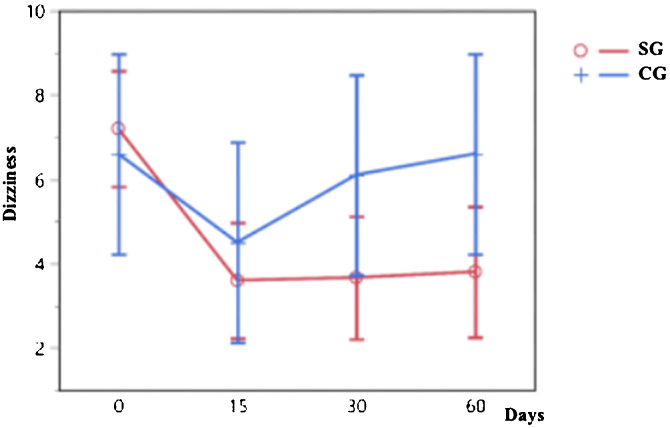
Progression of dizziness indices by VAS throughout the study, individualized for the SG (red line) and the CG (blue line) by the LSM.

The indices of dizziness reported by the VAS also presented a statistically significant difference between 0 and 15, 30 and 60 days in the SG (*p* < 0.0001), and not occurred in the CG (*p* = 0.3395) (Fig. 2).

### Muscle tension asymmetry

**Figure 3:**
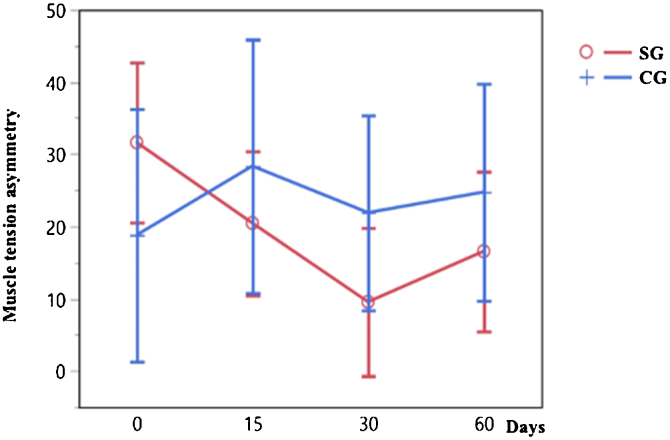
Progression of muscle tension asymmetry indices at zero, 15, 30, and 60 days in the SG (red line) and CG (blue line) by the Least Squares Mean (LSM).

There was a statistically significant difference in muscle tension asymmetry over time in the SG (*p* = 0.0165) between 0 and 30 days, which did not occur in the CG, as shown in Fig. 3.

### Evolution of pain, dizziness, and muscle tension asymmetry over time

The evolution of pain, dizziness, and muscle tension asymmetry over various time points for both the SG and the CG is shown in Table 2. The data illustrates the changes in these variables at baseline (time 0), as well as at subsequent intervals of 15, 30, and 60 days. The values in the table represent the mean scores for pain and dizziness experienced by participants in each group, as well as the muscle tension asymmetry (MTA) measurements for both groups. Notably, the *p*-values presented indicate the statistical significance of observed changes.

**Table 2:** Evolution of pain, dizziness, and muscle tension asymmetry over time.

Time	Pain (SG)	Pain (CG)	Dizziness (SG)	Dizziness (CG)	MTA (SG)	MTA (CG)
0	8.13	7.6	7.2	6.6	31.62	19.24
15	5.87	6.2	3.6	4.5	20.46	19.99
30	6.31	8	3.67	6.1	9.55	25.77
60	6.03	7.8	3.8	6.6	15.55	23.96
*p*-Value	<0.0001^a^	0.106	<0.0001^a^	0.3395	0.0165^a^	0.7155

CS, Control Group; SG, Study Group; MTA, muscle tension asymmetry.

a Significance level of 5% (*α* = 0.05) and a Confidence Interval of 95%.

### Imaging tests

There was no difference between imaging test findings between the groups. Statistical data and the main changes are described in Table 3.

**Table 3:** Frequency of imaging findings (XR, CT, and MRI) in the SG and CG.

Imaging findings	SG (n = 15)	CG (n = 5)	*p*
Uncovertebral arthrosis	8 (53%)	5 (100%)	0.11
Retrolisthesis	6 (40%)	3 (60%)	0.61
Anterior marginal osteophyte	5 (33%)	4 (80%)	0.13
Posterior marginal osteophyte	5 (33%)	3 (60%)	0.35
Intervertebral space reduction	5 (33%)	3 (60%)	0.35
Cervical lordosis rectification	4 (26%)	1 (20%)	1.00
Spondyloarthrosis	3 (20%)	2 (40%)	0.56
Intercalary bone	2 (13%)	2 (40%)	0.25
Diffuse bone demineralisation	1 (6%)	2 (40%)	0.14

CS, Control Group; SG, Study Group.

## Discussion

Neck pain is a prevalent symptom and is often associated with dizziness. The risk of falls and sequelae increased considerably when these patients are wrongly treated with depressant drugs.^14^

Most patients in this study are in their 6th decade of life, which is consistent with other studies on patients with CD.^15^ This fact can be attributed to the greater presence of muscle and joint degenerative processes inherent to ageing itself.^16^ Our sample was predominantly composed of women (90%). The lower percentage of males should be attributed to the fact that they consider themselves stronger, are resistant to treatment, and feel inferior when they are ill.^17^

Both groups had high levels of pain initially, which were higher in the SG. The effect of treatment during the study was evident in the SG but not in the CG. The mild pain reduction at the second assessment (day 15) in the CG can be attributed to the placebo effect itself, as the perception of pain increased again in later assessments. The decrease in pain perception demonstrates the effect of heat on the reduction of muscular tension, leading to diminished local contraction, vertebral tension, and pain. According to Mense, the contracted muscle presents ischemia and decreased pH, which sensitizes the nociceptors that trigger pain.^18^

Dizziness significantly improved in the SG between baseline and the following assessments. The same did not occur in the CG, which mimicked the results found for pain and showed no improvement. These observations show the association of pain and dizziness found in our study, corroborating previous studies.^13^, ^19^

As described for pain, muscle tension asymmetry was more intense in the SG, although not significantly. After treatment with local heat, asymmetry was significantly reduced in the SG at 15 and 30 days, increasing again on day 60 — one month from treatment ending. These findings demonstrate the heat's action, reducing the tension and asymmetry of the trapezius muscle during the intervention period with Helical® patches. Our results corroborate previous studies reporting the effectiveness of heat in reducing muscle and fascia tension^20^ showing the correlation between neck pain, dizziness, and muscle tension asymmetry.

Imaging tests showed similar degenerative cervical changes in both groups. Some authors attribute dizziness to the higher concentration of Ruffini’s corpuscle mechanoreceptors in the degenerated cervical discs of patients with CD.^21^

Based on our results, patients with CD show a clear interrelationship between pain, dizziness, and muscle tension asymmetry. Throughout the study, the improvement or worsening of one of these variables was always directly associated with the improvement or worsening of the others. As for treatment, thermotherapy was effective in controlling symptoms (pain and dizziness) up to one month after the end of the intervention. These results present a significant impact on clinical practice and point towards a promising direction for future research. The efficacy of nanotechnology in relieving pain and dizziness in patients diagnosed with CD has been established. The identification of asymmetry in muscle tension as a closely related factor to dizziness and pain clarifies some fundamental aspects for diagnosis. Due to its innocuous, practical, and straightforward application, nanotechnology emerges as an interesting approach for addressing cervical-origin dizziness.

The main limitations of the study lie in the selection of only 20 subjects with non-equitable distribution: 15 in the SG and five in the CG, as well as the short follow-up period, defined within 60 days. Nevertheless, rigorous selection criteria were employed, and these early and preliminary findings are promising. The benefits derived from this innovative treatment approach towards CD provide groundwork for more comprehensive investigations in the future.

## Conclusion

Dizziness is related to pain and asymmetry of muscular tension in the neck region. Heat from the CNT was effective in reducing dizziness in patients with CD.

## Funding

FAPESP ‒ Fundação de Amparo à Pesquisa do Estado de São Paulo ‒ Project 2016/20176-4.

## Conflicts of interest

The authors declare no conflicts of interest.
